# Outcomes of Weekday Versus Weekend Admissions for Heart Block Requiring De Novo Intracardiac Device Implantation

**DOI:** 10.7759/cureus.64141

**Published:** 2024-07-09

**Authors:** Abdel-Rhman Mohamed, Clarissa Pena, Sohrab Kadiver, Ahmad Abdelrahman, Omar Mousa, Ahmad Elzanaty, Blair Grubb

**Affiliations:** 1 Internal Medicine, The University of Toledo Medical Center, Toledo, USA; 2 Internal Medicine, Tanta University Faculty of Medicine, Tanta, EGY; 3 Cardiovascular Medicine, The University of Toledo Medical Center, Toledo, USA

**Keywords:** heart block, healthcare cost and utilization project, national inpatient sample, weekend, weekday, national inpatient sample (nis) and the healthcare cost and utilization project (hcup), cardiac device implantation, 3rd degree heart block

## Abstract

Even with comparable healthcare structure and staffing, patients presenting on weekends often face poorer outcomes, including longer wait times in the emergency department, extended hospital stays, and delays in major procedures. This discrepancy prompts questions about whether life-saving cardiac procedures, such as permanent pacemaker (PPM) implantation for atrioventricular block, also experience similar delays and differences in outcomes. We researched over 200,000 patients from the National Inpatient Sample (NIS) database to help study whether patients admitted on the weekend truly had worse outcomes than patients admitted on the weekday. Using the International Classification of Diseases, Tenth Revision (ICD-10) using STATA software (StataCorp LLC, College Station, TX), we found that 79.6% of patients were admitted on weekdays. Among these weekday admissions, 56.2% were males, with an average age of 75.8 years. Weekend admissions included 54.4% male patients, with an average age of 76.4 years. Key variables influencing outcomes were renal failure history, non-ST elevation myocardial infarction, diabetes mellitus, and percutaneous coronary intervention. Of the total patients, 1,315 died during hospitalization, with no significant difference in mortality between weekday and weekend admissions. However, weekend admissions had a higher rate of cardiac arrest, a greater likelihood of delayed pacer implantation, and longer hospital stays. Weekend admissions were linked to delays in PPM placement, longer hospital stays, and higher hospitalization costs. Mortality rates did not increase for patients admitted on weekends. Further research is needed to explore this issue in greater depth and to identify the specific factors contributing to the discrepancy between weekend and weekday admissions, which resulted in worse outcomes for weekend patients.

## Introduction

Despite similar healthcare structure and staff, there is an anecdote of poorer outcomes for acute patients presenting during the weekend. There is merit to some notions of sub-optimal patient experience regarding delays in undergoing major procedures, as described by Ryan et al., who showed that 36% of patients admitted on a weekend would immediately receive procedures, compared to 65% of patients admitted on a weekday [1]. This discrepancy prompts questions about whether life-saving cardiac procedures, such as permanent pacemaker implantation for atrioventricular block (AVB), also experience similar delays and differences in outcomes. AVB is the most common indication for permanent pacemaker implantation worldwide [2]. Permanent pacemaker implantation is almost always considered in patients with symptomatic second-degree AVB and third-degree AVB [3,4]. Looking at greater than 200,000 admissions for patients with heart block requiring de novo implantation of intracardiac devices, we aim to examine differences in inpatient mortality, length of hospitalization, and total hospital charge for patients admitted on a weekend versus weekday.

## Materials and methods

This is a retrospective observational study of patients aged 18 years or older. The National Inpatient Sample (NIS) 2016-2020 was queried for adult hospitalizations for heart block requiring de novo intracardiac device implantation. Hospitalizations were stratified as weekend vs. weekday admissions. Data were collected using the NIS through the Healthcare Cost and Utilization Project (HCUP). The primary diagnosis was identified using the International Classification of Diseases, Tenth Revision (ICD-10) codes. The study was not reported using the STROBE (Strengthening the Reporting of Observational Studies in Epidemiology) guidelines. Multivariable regression analyses were used to determine the association between weekend admission and hospitalization outcomes. Outcomes evaluated were in-hospital mortality, cardiac arrest, the likelihood of delayed pacer implantation, and the likelihood of prolonged length of stay (LOS). Delayed pacing was defined as time to pacer implantation greater than 75% percentile of the whole cohort. Prolonged LOS was also defined as LOS greater than 75% percentile of the whole cohort. Of the patients who fit our criteria, we identified other comorbidities and diagnoses associated with them as potential confounders, including hypertension, diabetes, obesity, rheumatoid disease, peripheral vascular disease, liver disease, renal disease, history of coronary artery bypass graft (CABG), myocardial infarction (MI), and percutaneous coronary intervention (PCI), as well as chronic lung disease. Other confounders that were adjusted for include race, Charlson Comorbidity Index, median household income, insurance, hospital region in the US, hospital bed size, and teaching status of the hospital.

Multivariable regression analyses were used to determine the association between weekend versus weekday on inpatient mortality, length of hospitalization, and cost of hospitalization of patients admitted for de novo intracardiac device implantation. The statistical analysis was carried out using STATA version 17.0 software (StataCorp LLC, College Station, TX). The data for categorical variables were expressed as percentages, whereas for continuous variables, mean ± standard deviation (SD) was used. Student's t-test was used for comparing continuous variables, while the chi-square test was used for categorical variables. For the primary and secondary outcomes, unadjusted odds ratios were calculated using univariate regression analysis. To adjust for potential confounders and calculate adjusted odds ratios (aOR), multivariate regression analysis was used. Binary outcomes were analyzed using a logistic regression model, while continuous outcomes were analyzed using linear regression. The models were constructed by incorporating the variables associated with the outcome of interest in the univariable regression analysis, with a cut-off p-value of 0.02. All p-values were two-tailed, and the threshold for statistical significance was set at 0.05.

## Results

The number of patients analyzed from the NIS database was 201,410. Of those patients, about 79.6% were admitted during a weekday. Of patients who were admitted on a weekday, the majority of patients were male (56.2%). The average age of patients admitted on a weekday was 75.8 ± 10.8 years. Of patients who were admitted on a weekend, the majority of patients were male (54.4%). The average age of patients admitted on the weekend was 76.4 ± 10.4 years (Table 1). Statistically significant variables included history of renal failure, non-ST elevation myocardial infarction (NSTEMI), diabetes mellitus, and PCI.

**Table 1:** Patient demographics and comorbidities of weekday versus weekend admission for intracardiac device implantation for complete heart block. STEMI: ST-segment elevation myocardial infarction; NSTEMI: non-ST elevation myocardial infarction; CABG: coronary artery bypass graft; MI: myocardial infarction; PCI: percutaneous coronary intervention.

Patient demographics and comorbidities	Admitted on a weekday	Admitted on a weekend	p-value
Number of patients	160,280 (79.6%)	41,130 (20.4%)	-
Mean age (SD)	75.8 ± 10.8	76.4 ± 10.4	<0.001
Female	70,205 (43.8%)	18,750 (45.6%)	0.003
Hypertension	135,135 (84.3%)	34,865 (84.8%)	0.317
Third-degree AV block	117,665 (73.4%)	30,500 (74.2%)	0.172
Second-degree AV block	40,440 (25.2%)	10,005 (24.3%)	0.090
First-degree AV block	2,175 (1.4%)	625 (1.5%)	0.256
Diabetes mellitus	59,275 (37.0%)	15,785 (38.4%)	0.019
Atrial fibrillation	27,350 (17.1%)	7,315 (17.8%)	0.128
STEMI	415 (0.3%)	130 (0.3%)	0.359
NSTEMI	3,525 (2.2%)	1,265 (3.1%)	<0.001
Charlson Comorbidity Index			-
0	38,390 (24.0%)	9,155 (22.3%)	
1	38,955 (24.3%)	9,480 (23.0%)	
2	28,795 (18.0%)	7,620 (18.5%)	
3+	54,140 (33.8%)	14,875 (36.1%)	
Smoking	14,035 (8.8%)	3,520 (8.6%)	0.567
Active cancer	5,415 (3.4%)	1,480 (3.6%)	0.326
Congestive heart failure (CHF)	50,305 (31.4%)	13,285 (32.3%)	0.113
Peripheral vascular disease (PVD)	13,060 (8.1%)	3,210 (7.8%)	0.311
Rheumatoid disease	4,645 (2.9%)	1,290 (3.1%)	0.254
Hypothyroidism	30,055 (18.8%)	8,065 (19.6%)	0.078
Renal failure	42,365 (26.4%)	11,400 (27.7%)	0.019
Liver disease	3,695 (2.3%)	1,060 (2.6%)	0.151
Lymphoma	1,125 (0.7%)	255 (0.6%)	0.419
Rheumatoid arthritis/collagen vascular	5,155 (3.2%)	1,400 (3.4%)	0.390
Obesity	27,575 (17.2%)	7,210 (17.5%)	0.481
Valvular disease	35,645 (22.2%)	9,330 (22.7%)	0.388
Pulmonary circulation disorders	11,500 (7.2%)	2,975 (7.2%)	0.859
Chronic pulmonary disease	30,625 (19.1%)	8,135 (19.8%)	0.169
History of CABG	16,135 (10.1%)	4,235 (10.3%)	0.542
History of MI	13,925 (8.7%)	3,590 (8.7%)	0.908
History of PCI	16,890 (10.5%)	4,695 (11.4%)	0.020
History of stroke	14,325 (8.9%)	3,620 (8.8%)	0.704
Race (uniform)			0.004
White	120,855 (77.7%)	31,405 (78.7%)	
Black	14,040 (9.0%)	3,065 (7.7%)	
Hispanic	12,320 (7.9%)	3,220 (8.1%)	
Asian or Pacific Islander	3,590 (2.3%)	1,035 (2.6%)	
Native American	760 (0.5%)	170 (0.4%)	
Other	3,995 (2.6%)	1,000 (2.5%)	
Median household income national quartile for patient ZIP code			0.661
0-25th percentile	38,850 (24.6%)	9,970 (24.6%)	
26th to 50th percentile (median)	41,215 (26.1%)	10,830 (26.7%)	
51st to 75th percentile	41,000 (25.9%)	10,315 (25.4%)	
76th to 100th percentile	36,970 (23.4%)	9,475 (23.3%)	
Primary expected payer (uniform)			0.199
Medicare	126,750 (79.1%)	32,925 (80.1%)	
Medicaid	5,300 (3.3%)	1,350 (3.3%)	
Private insurance	22,480 (14.0%)	5,495 (13.4%)	
Self-pay	2,050 (1.3%)	540 (1.3%)	
No charge	145 (0.1%)	20 (0.0%)	
Other	3,450 (2.2%)	760 (1.8%)	
Region of hospital			0.227
Northeast	34,570 (21.6%)	8,495 (20.7%)	
Midwest	37,540 (23.4%)	9,670 (23.5%)	
South	57,240 (35.7%)	14,735 (35.8%)	
West	30,930 (19.3%)	8,230 (20.0%)	
Relative bed size category of hospital (Strata)			0.697
Small	25,630 (16.0%)	6,475 (15.7%)	
Medium	47,290 (29.5%)	12,315 (29.9%)	
Large	87,360 (54.5%)	22,340 (54.3%)	
Location/teaching status of hospital (Strata)			0.410
Rural	8,845 (5.5%)	2,115 (5.1%)	
Urban non-teaching	33,345 (20.8%)	8,650 (21.0%)	
Urban teaching	118,090 (73.7%)	30,365 (73.8%)	

The number of patients who died during hospitalization was 1,315, and there was no statistical difference in mortality between days of admission (mortality = aOR: 1.01; CI: 0.74-1.37; p = 0.95). Patients admitted on a weekend had a statistically higher rate of cardiac arrest (aOR: 1.29; CI: 1.13-1.47; p < 0.01), higher likelihood of delayed pacer implantation (aOR: 2.83; CI: 2.68-3.00; p < 0.01), and higher chances of prolonged hospitalization (aOR: 1.4; CI: 1.33-1.48; p < 0.01) (Table 2).

**Table 2:** Patient outcomes of weekday versus weekend admission for intracardiac device implantation for complete heart block.

Outcomes	Admitted on a weekday	Admitted on a weekend	p-value
Number of patients	160,280 (79.6%)	41,130 (20.4%)	-
Died during hospitalization	1010 (0.6%)	305 (0.7%)	0.260
Received blood transfusion	1850 (1.2%)	400 (1.0%)	0.160
Pneumothorax	2130 (1.3%)	505 (1.3%)	0.480
Cardiac tamponade	470 (0.3%)	80 (0.2%)	0.127
Cardiac arrest	5173 (3.2%)	1745 (4.2%)	<0.01
Delayed pacing	53,320 (33.3%)	23,855 (58.0%)	<0.01
Prolonged length of stay (LOS)	56,715 (35.4%)	17,630 (42.9%)	<0.01
Total cost	$31,540.30 ± $33,387.50	$34,132.60 ± $34,246.70	<0.01
Came from skilled nursing facility (SNF), or other short-term care, home-care	49,225 (30.7%)	14,560 (35.4%)	<0.01
Length of stay	3 (2-4)	3 (2-5)	<0.01

## Discussion

In this nationwide observational study, 201,410 patients were admitted with heart block requiring de novo implantation of intracardiac devices. We aimed to evaluate the impact of weekend vs. weekday admission on outcomes. Our study determined several outcomes of importance. First, there was no difference in mortality between the weekday and weekend cohorts. Second, there was a greater delay in intracardiac device placement with the weekend cohort of patients. Third, a greater proportion of the weekend cohort was found to have increased rates of cardiac arrest.

AVB is caused by the delay or blockade of the electrical impulse from the atria to the ventricles [5]. This could be due to functional or anatomical abnormalities in the heart’s conduction system [6]. Third-degree block, also known as complete heart block, implies that there is no conduction between the atria and the ventricles, which, if not treated, can lead to sudden cardiac death [7]. However, heart block can sometimes be reversible and require only temporary pacing until the underlying cause is properly treated [8-11]. Various causes of reversible complete AVB have been reported, including myocardial ischemia, cardiac surgery, drugs, toxins, Lyme disease, hypothyroidism, and metabolic abnormalities, such as hyperkalemia [12-14]. In an emergency setting, some parameters could be used to assess the need for a permanent pacemaker (PPM) placement, such as male gender, systolic blood pressure, left ventricular ejection fraction, and pre-admission β-blocker use [15]. However, implant-related complications are possible. Pacemaker implantation requires venous puncture that may, due to its proximity to vital structures, cause pneumothorax and hemothorax [16]. Patients treated with anticoagulant and antiplatelet drugs may be at increased risk of developing pocket hematoma [17].

The weekend effect has been described by prior studies whereby there is a higher incidence of mortality seen among patients admitted over the weekend. Several of these conditions were thought to be emergent conditions that would have conferred a high mortality outside of a critical care setting [18]. Abrich et al. performed a retrospective observational study of two centers that found there were no significant differences in mortality between patients who had pacemakers implanted on the weekend vs. weekdays [19]. In the current study, as there was no difference in the proportion of patients admitted with varying degrees of AVB, it is reasonable to assume there was little difference in the urgency of PPM placement. Viewing mortality through this context may explain why there is no difference in mortality between the two cohorts.

Within the context of the weekend effect and its impact on morbidity and mortality in cardiac patients, the phenomenon is not unique to electrophysiology patients. Kostis et al. identified a convincing increase in mortality for patients with myocardial infarction admitted on weekends [20]. Associated with these weekend admissions was lower use of invasive cardiac procedures, which was identified as a cornerstone of this increase in mortality. Kostis et al. cited the National Registry of Myocardial Infarction database whose data confirmed off-hour presentation in the setting of myocardial infarction was linked with delay in implementation of primary percutaneous intervention, which itself translated to higher mortality.

Regarding delayed pacing being greater in patients admitted over the weekend, this may be due to the unavailability of services during the weekend, most notably procedure room availability and staffing. It is well-documented that decreased staffing is associated with poorer patient outcomes [21,22]. Pronovost et al. describe that with high-intensity physician staffing, 94% of the studies they reviewed reported a decrease in hospital mortality, 93% of the studies showed a decrease in ICU mortality, 77% of the studies showed a decrease in hospital LOS with high-intensity physician staffing, and 78% of the studies showed a decrease in ICU LOS with high-intensity physician staffing [21]. High-intensity physician staffing was defined as closed ICU or mandatory intensivist consultation, while low-intensity physician staffing was defined as no intensivist on the case or elective consultation. The limitation of this study is that it was designed specifically for an ICU setting. On the other hand, Needleman et al. describe that with lower levels of staffing of registered nurses (RNs), there was an overall increase in patient mortality. They received data from 2003 to 2006 on 197,961 admissions from 43 hospitals. They found higher rates of hospital mortality with high-turnover shifts and below-target shifts [22]. These shifts were defined as follows: the difference between target RN hours for the shift and actual hours worked on the unit in direct patient care was calculated, and below-target staffing was defined as eight hours or more below the adjusted target. They constructed a measure of patient turnover equal to the sum of unit admissions, transfers, and discharges, as well as the adjusted census at the beginning of their shift; and high turnover was defined as if the rate was greater than or equal to the mean plus 1 SD. Greater delay of PPM placement in the weekend cohort would directly confer greater LOS and hospitalization costs in the present study. These findings are consistent with prior literature demonstrating that the presence of less high-intensity staff was associated with greater LOS [21,23].

A greater proportion of patients suffered cardiac arrest in the weekend cohort. The association between high-degree AVB and cardiac arrest has been documented [24]. Our study does not show a statistically significant difference between the subtypes of heart block making this less likely as an explanation for why more patients on the weekend suffered cardiac arrest. It is more likely that the weekend cohort suffered cardiac arrest in greater proportion due to being a sicker patient population. Evidence of this claim is supported by the weekend cohort being more statistically likely to have NSTEMI, diabetes mellitus, renal failure, and a history of PCI.

Limitations

Our study findings should be interpreted in the context of its limitations. First, due to the utilization of a database that relies on coding, the study is prone to documentation errors. Second, it is unknown if these admissions were elective, urgent, or emergent. Third, we do not know the cause of each bradyarrhythmia. Fourth, though we adjusted for confounders that might impact PPM timing in the multivariate regression model, there is a lack of information about center-specific information like electrophysiology lab, equipment, and staff availability that impact the timing of the PPM.

## Conclusions

Weekend admissions were associated with delayed PPM placement, greater LOS, and increased hospitalization costs. Mortality was not increased among patients admitted over the weekend. Further research is required to investigate this topic further. It is necessary to identify the specific factors that may have caused a discrepancy between weekend and weekday admissions that led to worse outcomes in those specific patient populations.
